# Disorders of Sex Development and Hypogonadism: Genetics, Mechanism, and Therapies

**DOI:** 10.1155/2012/820373

**Published:** 2012-07-19

**Authors:** Gil Guerra-Junior, Ana Claudia Latronico, Olaf Hiort, Rodolfo Rey

**Affiliations:** ^1^Pediatric Endocrinology Unit, Department of Pediatrics, Faculty of Medical Sciences, State University of Campinas (UNICAMP), Campinas, SP, Brazil; ^2^Lab of Hormones and Molecular Genetics (LIM/42), Endocrinology of Development Unit, School of Medicine, University of São Paulo (USP), São Paulo, SP, Brazil; ^3^Division of Paediatric Endocrinology and Diabetes, Department of Pediatric and Adolescent Medicine, University of Lübeck, Germany; ^4^Division of Endocrinology, Center of Endocrinology Investigation (CEDIE-CONICET), Children Hospital Dr. Ricardo Gutiérrez, Buenos Aires, Argentina

Disorders of sex development (DSD) and hypogonadism are genetically heterogeneous and include a broad spectrum of phenotypes. Recent advances in biology and medicine have introduced impressive improvements in both clinical management and structured research, mainly in new technologies to study their genetic features and the mechanisms underlying their pathologies. Knowledge and understanding of these conditions have led to the development of successful therapies and novel tools to characterize them and provide better care to patients. Therefore, both DSD and hypogonadism represent an important field in research and clinical setting. 

This *special issue* presents a series of articles mainly reflecting the difficulties in characterization of DSD in four papers, advances in genetics of DSD in two papers, hypogonadotropic hypogonadism in one paper, clinical presentation of male primary hypogonadism, like Klinefelter's syndrome in one paper, and long-term followup of female hypergonadotropic hypogonadism in one paper, as well as clinical and molecular data of aromatase excess syndrome in one paper and an interesting review about the complex association of androgens and adipose tissue in males in one paper. 

Juniarto and colleagues in their paper “Application of the new classification on patients with a disorder of sex development in Indonesia” from Indonesia showed how patients' and general society's opinion on DSD, economic background of the patients, and lack of access to health insurance can affect the complex management of DSD in a negative way. Probably, these situations are not restricted to Indonesia, but to all underdeveloped countries, and education of primary health care workers is necessary to prevent morbidity and mortality in some cases. Hersmus and colleagues in their paper “Delayed recognition of disorders of sex development (DSD): a missed opportunity for early diagnosis of malignant germ cell tumors” reported three independent male patients with germ cell tumors retrospectively recognized as DSD, showing again, but now in a developed country, the underdiagnosis of DSD in the newborn and the significant relevance of early and correct evaluation for identification of individuals at increased risk for development a gonadal tumor. Wünsch's paper “Checklist for the structural description of the deep phenotypes in disorders of sexual development” suggest an interesting checklist for the structural description of gonads and internal sex ducts using video endoscopy and laparoscopy, which can contribute to a comprehensive definition of the variations of deep phenotype in DSD. Veiga-Junior and colleagues in their paper “Clinical and laboratorial features that may differentiate 46,XY DSD due to partial androgen insensitivity and 5*α*-reductase type 2 deficiency”, evaluating 58 patients with sex ambiguity, 46,XY karyotype, and normal testosterone secretion, reported clinical and laboratorial features, like consanguinity, familial recurrence, severity of ambiguous genitalia, penile length, and T/DHT ratio, which may differentiate 46,XY DSD due to partial androgen insensitivity and 5*α*-reductase deficiency.

Barbaro and colleagues in their paper “Multigeneration inheritance through fertile XX carriers of an *NR0B1 *(*DAX1*) locus duplication in a kindred of females with isolated XY gonadal dysgenesis” identified another *NR0B1 *duplication in two sisters with isolated XY gonadal dysgenesis and an X-linked inheritance pattern. They also studied three fertile female carriers of three different small *NR0B1 *locus duplications and did not find obvious skewing of X-chromosome inactivation, suggesting that *NR0B1 *overexpression does not impair ovarian function. Mooslehner and colleagues in their paper “A cell model for conditional profiling of androgen-receptor-interacting proteins” reported preliminary results that allow future studies to focus on replacing wild-type androgen receptor (AR) with mutant AR to uncover differences in protein interactions caused by AR mutations involved in partial androgen insensitivity syndrome. 

Beate and colleagues in their paper “Genetics of isolated hypogonadotropic hypogonadism: role of GnRH receptor and other genes” showed an interesting revision about the genetics of isolated hypogonadotropic hypogonadism and the importance of inactivating mutations in the pituitary GnRH receptor inducing GnRH resistance in patients with normosmic isolated hypogonadotropic hypogonadism. Pacenza and colleagues in their paper “Clinical presentation of Klinefelter's syndrome: differences according to age”, evaluating 94 patients with Klinefelter's syndrome, showed that 46.8% were diagnosed between 11 and 20 years, probably because nowadays pediatricians are more aware that boys and adolescents with neurodevelopmental disorders and cryptorchidism should be investigated for this chromosome anomaly. Pantelis and colleagues in their paper “Long-term followup of adolescent and young adult females with hypergonadotropic hypogonadism” showed that all prepubertal girl, teenager, or young woman diagnosed with primary ovarian insufficiency should undergo extensive research by a group of specialists in a referral center, and the final decision of therapy should be taken by the patient and her clinician. 

Fukami and colleagues in their paper “Molecular bases and phenotypic determinants of aromatase excess syndrome” showed that aromatase excess syndrome represents a novel model for gain-of-function mutation leading to human genetic disorders characterized by gynecomastia. Mammi and colleagues in their paper “Androgens and adipose tissue in males: a complex and reciprocal interplay” reported an interesting revision about the association between androgens and adipose tissue in males. The authors concluded that adequate levels and balance of circulating sex hormones are necessary to maintain a correct distribution and size of adipose tissue, which in turn is fundamental to keep a normal reproductive and sexual function. 

Our understanding in DSD and hypogonadism is advancing, and we hope that the readers will find this *special issue *enjoyable and helpful in the clinical practice. In addition, we would like to take the opportunity to express our gratitude and thanks to all the authors involved in this selected issue for the high-level papers they submitted. We also thank the reviewers for their time and expertise as well as the Editor-Chief of International Journal of Endocrinology for giving us the honor to publish this important *special issue*. Enjoy this *special issue* of DSD and hypogonadism! 

*Gil Guerra-Junior* *Gil Guerra-Junior* *Ana Claudia Latronico* *Ana Claudia Latronico* *Olaf Hiort* *Olaf Hiort* *Rodolfo Rey* *Rodolfo Rey*

